# Hypoxic Signalling in Tumour Stroma

**DOI:** 10.3389/fonc.2018.00189

**Published:** 2018-05-29

**Authors:** Anu Laitala, Janine T. Erler

**Affiliations:** Biotech Research and Innovation Centre (BRIC), University of Copenhagen (UCPH), Copenhagen, Denmark

**Keywords:** hypoxia, HIF, PHDs, tumour stroma, metastasis

## Abstract

Hypoxia is a common feature in solid tumors and is associated with cancer progression. The main regulators of the hypoxic response are hypoxia-inducible transcription factors (HIFs) that guide the cellular adaptation to hypoxia by gene activation. The actual oxygen sensing is performed by HIF prolyl hydroxylases (PHDs) that under normoxic conditions mark the HIF-α subunit for degradation. Cancer progression is not regulated only by the cancer cells themselves but also by the whole tumor microenvironment, which consists of cellular and extracellular components. Hypoxic conditions also affect the stromal compartment, where stromal cells are in close contact with the cancer cells. The important function of HIF in cancer cells has been shown by many animal models and described in hundreds of reviews, but less in known about PHDs and even less PHDs in stromal cells. Here, we review hypoxic signaling in tumors, mainly in the tumor stroma, with a focus on HIFs and PHDs.

## Introduction

Solid tumors are most often partly hypoxic. During the rapid growth of cancer cells, the surrounding vasculature becomes inadequate and is unable to meet the high demand of oxygen creating heterogeneously distributed hypoxic areas within the tumor. Hypoxia in tumors promotes abnormal angiogenesis, desmoplasia, and inflammation. It also boosts the selection of cancer cells that have a more malignant phenotype promoting tumor progression and metastasis, and thereby serving as an indicator for disease outcome. Hypoxic tumor cells are also resistant to radiotherapy and most chemotherapies. Tumors include stromal cells and extracellular matrix (ECM) in addition to cancer cells, which are also affected by the hypoxic environment (1, 2). An ever-increasing number of studies have highlighted the importance of the tumor microenvironment (TME) in regulating tumor progression and dissemination (3).

The cellular response to the drop in oxygen concentration leads to stabilization of hypoxia-inducible transcription factor (HIF) in all cell types, which is one of the key regulators of hypoxia response. The HIF prolyl hydroxylases (PHDs) are oxygen-dependent enzymes that target HIF for degradation in air. Thus, in hypoxia, PHDs are inactive, and HIF is stabilized. HIF regulates the expression of many genes that help cells to adapt to hypoxic conditions by decreasing oxygen consumption and increasing its supply. Generally, this includes shifting the energy metabolism to the less oxygen requiring glycolytic pathway and stimulation of the angiogenic genes to increase vascular flow to the hypoxic regions (4).

In addition to HIF activation, responses to hypoxia are also mediated through HIF-independent pathways. These signaling mechanisms include the unfolded protein response (UPR) and mammalian target of rapamycin (mTOR) signaling (5). They work as independent pathways, but in many cases, their signaling is integrated with HIF activation as well as with each other (6).

Unfolded protein response is activated during endoplasmic reticulum (ER) stress. ER stress arises from accumulation of unfolded proteins, which can be contributed by several factors such as nutrient and calcium depletion and hypoxia. It consists of different and complex signal-transduction cascades, where pancreatic ER kinase (PERK), inositol-requiring enzyme 1, and activating transcription factor 6 act as sensor proteins, situated in the ER membrane. These mediate pro-survival signaling aiming to counteract ER stress by limiting the production of unfolded proteins and degrading the misfolded proteins preventing cellular damage (5, 7). However, if UPR is not able to re-establish ER homeostasis, it can also lead to pro-death signaling leading to apoptosis. Many publications have shown UPR to be activated in numerous tumor types where hypoxia is one of the promoting factors. Cancer cells have, however, found ways to avoid the ER stress-induced apoptosis (7). UPR signaling has been shown to target vascular endothelial growth factor (VEGF) in cancer cells. Activation of transcription factor 4, which is downstream of the PERK, was shown to induce VEGF mRNA levels, but to lesser extent than HIF signaling. UPR was, nevertheless, also shown to phosphorylate HIF-1α enhancing its activity, which resulted in increased VEGF expression (8). URP signaling has also been shown to have a role in tumor infiltrating immune cells, which can promote both immunosurveillance and immune escape mechanisms (9).

The mTOR kinase is a part of the mTOR complexes 1 and 2 (mTORC1 and 2), and it regulates cell survival, growth, and metabolism. mTOR mediates signals from different intracellular and extracellular stimuli such as growth factors, nutrients, stress, and oxygen levels (10). As a result, mTOR is phosphorylated at multiple sites, which in turn leads to direct phosphorylation of downstream targets such as eukaryotic translation initiation factor 4E binding protein 1 and ribosomal protein S6 kinase. Many of mTOR upstream signaling pathways go through the tumor suppressor tuberous sclerosis 1 and 2 (TSC1/2) complex, and it is also subject to hypoxic regulation. TSC1/2 complex negatively regulates the small GTPase ras-homolog-enriched-in-brain (Rheb). During mTOR activating signaling, TSC1/2 complex is inhibited by phosphorylation, leading to mTOR activation through active Rheb (6, 11). Hypoxia is known to increase the ratio of AMP/ATP, which leads to activation of AMP-activated protein kinase (AMPK). AMPK is capable of phosphorylating TSC2, but this phosphorylation on the contrary activates the TSC1/2 complex, which inhibits Rheb. Inactive Rheb in turn cannot phosphorylate mTOR, which attenuates the downstream signaling (12). mTOR signaling also intercepts with HIF-dependent hypoxic signaling. HIF upregulates the expression of REDD1 (regulated in development and DNA damage responses), which also activates TSC1/2 complex decreasing mTOR signaling (13). However, this inhibitory mechanism seems to have cell type-dependent differences (14). Oncogenic mTOR has also been reported to promote HIF signaling as well as HIF target gene expression such as lysyl oxidase (LOX) (15) in an HIF-dependent way. VEGF, however, has been shown to be regulated by both HIF-dependent and HIF-independent mechanisms (16). This was also seen in cancer-associated fibroblasts (CAFs) isolated from human breast cancers where tumor suppressor p16^INK4A^ downregulation led to increased Akt/mTOR signaling also affecting HIF-α positively and further increasing the VEGF-A secretion. Enhanced Akt/mTOR signaling in CAFs was shown also to increase their invasion and migration capabilities. However, the role of HIF was not addressed (17).

Of these three different hypoxia response pathways mentioned, we will focus on the HIF signaling pathway that influences both cancer and stromal cells. Little is known about how HIF/PHDs activities regulate stromal cell function in the TME. Here, we review how HIF/PHD pathways impact on cancer progression in the different cellular and non-cellular compartments of TME.

## HIFs and PHDs: Key Players in Oxygen Sensing and Hypoxia Signaling

Hypoxic signaling is mediated *via* the hypoxia-inducible transcription factor (HIF), which is a dimer consisting of two subunits, HIF-α and HIF-β. HIF-β (also known as ARNT) is constitutively expressed and stabile, but HIF-α is targeted to proteasomal degradation under normoxic conditions. Both subunits contain basic helix-loop-helix and PER-ARNT-SIM domains that are important for DNA binding and dimerization, but only HIF-α contains an oxygen-dependent degradation (ODD) domain that is important for the oxygen-dependent regulation. Under hypoxic conditions, HIF-α is able to bind with HIF-β forming a dimer that specifically binds to hypoxia response elements and so activates the transcription of over hundreds of genes that help the cells to adapt to low oxygen levels (18–21).

HIF-α has three different isoforms, HIF-1α, HIF-2α, and HIF-3α, of which the two first are more widely studied. HIF-1α and HIF-2α resemble each other, but the main differences are in their N-terminal transactivation domain (N-TAD). They both have a similar C-terminal transactivation domain (C-TAD), which contributes to the transcription of their shared targets, whereas the N-TAD differences confer the different binding capabilities and specificity among their targets (22). HIF-3α is the most different from the three and is subject to extensive alternative splicing resulting in many splicing variants. It has only one specific proline residue in the ODD domain and is lacking the C-TAD. Its function only as a transcription factor has been doubted and in fact different HIF-3α variants have been shown to have inductive and suppressive effects on HIF targets (23, 24).

**Figure 1:**
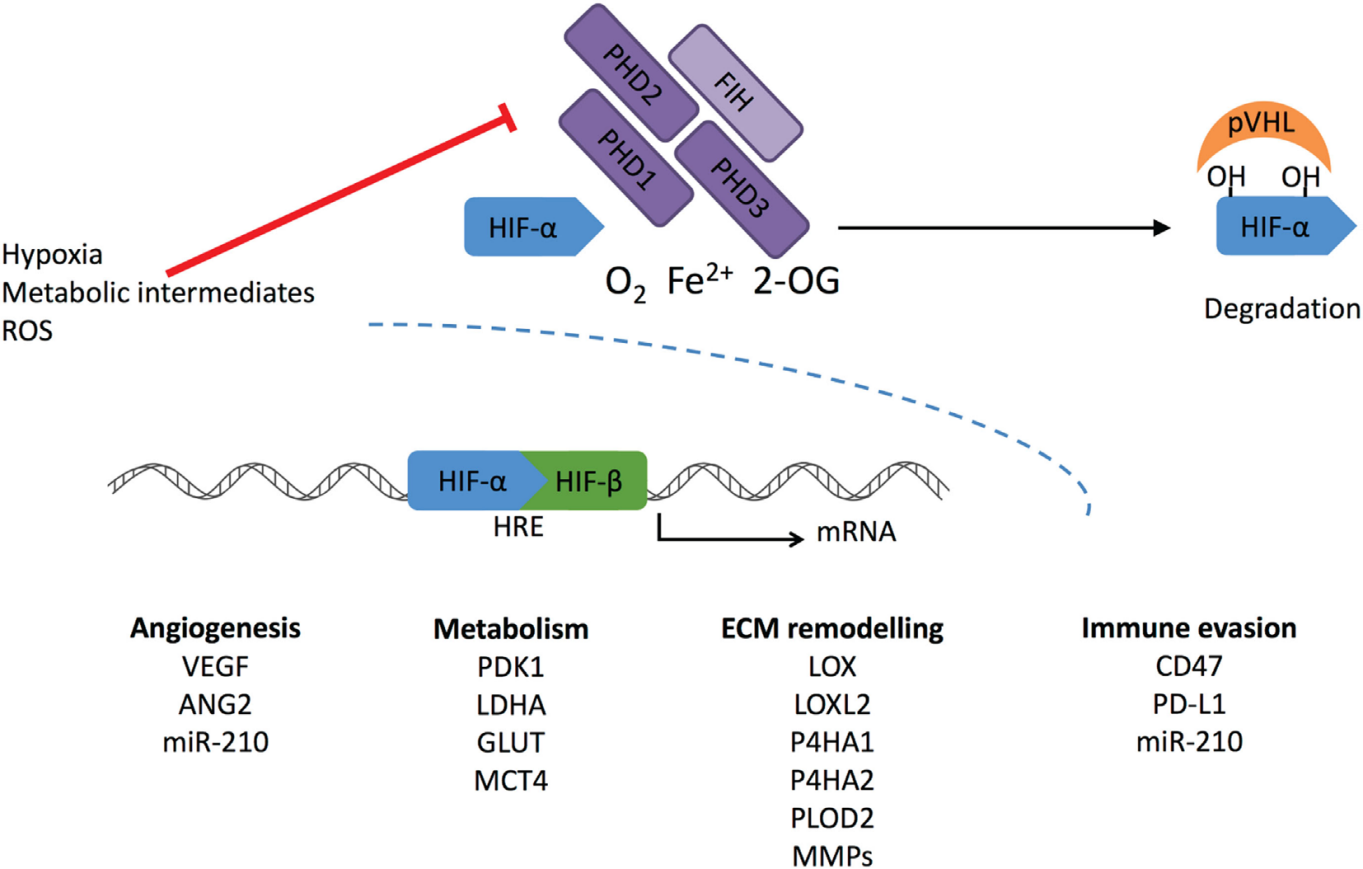
Regulation of HIF-α stabilization. Cellular oxygen levels are sensed by HIF prolyl hydroxylase (PHD) 1–3. They hydroxylate two specific proline residues in the HIF-α subunit, which require O_2_, Fe^2+^, and 2-oxoglutarate (2-OG). 4-Hydroxyprolines are recognized by von Hippel-Lindau (pVHL) complex that ubiquitylates HIF-α causing its proteasomal degradation. Another member of the dioxygenase superfamily, factor-inhibiting HIF (FIH), hydroxylates one asparaginyl residue at the transcriptional domain of HIF causing suppression of the transcriptional activity. Under hypoxic conditions, PHD and FIH activity is inhibited, and unhydroxylated HIF-α forms a complex with HIF-β and activates transcription of HIF target genes. Reactive oxygen species (ROS) and intracellular metabolites can inhibit PHDs and FIH-stimulating transcriptional activation of HIF-1α, leading to increased expression under normoxic conditions.

The ODD domain contains two specific proline residues (Pro402 and 564 in human HIF-1α) that are hydroxylated under normoxic conditions by the HIF prolyl hydroxylase 1–3 (also known as EGLN2, 1, and 3 or HIF-P4H1-3) (Figure 1). These enzymes belong to the 2-oxoglutarate (2-OG)-dependent dioxygenases and require oxygen, iron, ascorbate, and 2-OG for the hydroxylation reaction. The hydroxylated prolines act as recognition sites for the von Hippel-Lindau (pVHL)–E3 ubiquitin ligase complex, which tags HIF-α for proteasomal degradation (18, 25, 26). HIF is also regulated by factor-inhibiting HIF (FIH) in normoxia. FIH belongs to the same 2-OG family as PHDs. It hydroxylates the asparagine residue within the C-TAD blocking HIF from binding to the p300-CBP coactivators and inhibits the transcriptional activation of HIF (27).

HIF prolyl hydroxylases are fast in responding are fast in responding to changes in O_2_ concentration due to their much higher *K*_m_ values than the physiological O_2_ concentrations (28). They are widely expressed in different tissues, but at varying levels. PHD2 is the main enzyme to regulate HIF. Inactivation of PHD2 alone leads to HIF stabilization in normoxia. Both PHD1 and PHD3 are generally regarded to have a complementary role as inactivation of these alone does not lead to HIF stabilization (29). Global inactivation of PHDs in mice supports the finding that PHD2 is a key modulator in HIF regulation. Inactivation of PHD2 led to embryonic lethality, whereas phenotypes resulting from PHD1 or PHD3 inactivation were viable and grossly normal (30). PHDs have specificity toward different HIF-α hydroxylations. PHD2 preferentially hydroxylates HIF-1α, whereas PHD3 has a preference toward HIF-2α (31). Both PHD2 and PHD3 are hypoxia inducible, and it seems PHD2 is upregulated more by HIF-1 and PHD3 by HIF-2 (32). PHD2 has been reported to bind HIF-1α also in hypoxia inhibiting the HIF-1α N-terminal transcriptional activity without affecting its proteolysis (33).

HIF prolyl hydroxylases have been reported to have additional targets and to participate in HIF-independent signaling. PHD2 has been shown to inhibit Akt by hydroxylation and binding of pVHL. As hypoxia inhibits PHD2, it activates and promotes tumor growth *via* Akt (34). PHD3 has been reported to interact with activating transcription factor-4, which has been shown to activate genes involved in redox balance, apoptosis, and general cell survival under conditions of compromised nutrition (35, 36). PHD3 has been associated with apoptosis *via* different mediators. For example, PHD3 induces a neuronal regulator kinesin family member 1B β (KIF1β) that in turn induces apoptosis. For this interaction, PHD3 hydroxylation activity was required, but it is still not known whether there is direct hydroxylation of KIF1β (37). PHD3 has been found to hydroxylate and activate the human homolog of the *Caenorhabditis elegans* biological clock protein CLK-2 (HCLK2), which is an important player in DNA damage response. This was required for the activation of the ATR/CHK1/p53 pathway, so that inhibition of PHD3 attenuated DNA damage and the consequent apoptosis (38). PHD3 also directly inactivates the β2-adrenergic receptor (β_2_AR) by hydroxylating the intracellular domain, which leads to binding of the pVHL and subsequent degradation. β_2_AR activation is also known to play a role in apoptosis (39, 40).

## HIF and PHDs in Tumorigenesis: Bad Cops?

Extensive literature exists regarding the role of HIF and PHDs expressed by tumor cells. We will only touch on a few aspects here, and we recommend more in-depth reviews such as Rankin et al. (41) and Schito and Semenza (4) for more detailed information. Generally, HIF activation in cancer cells has been regarded as tumor promoting. In solid tumors, this usually results from oxygen deprivation leading to hypoxia. However, HIF signaling can also be activated by other oncogenic or tumor suppressor pathways, but mutations directly affecting HIF activation are not common in cancer, with the exception of pVHL deficiency (42). As pVHL mediates the proteolysis of HIF-α subunits, inactivation of this protein leads to constitutively active HIF also in normoxic conditions. However, Von Hippel-Lindau mutations are responsible only for a limited set of cancers such as clear cell renal cancer (ccRCC), pheochromocytoma, and pancreatic neuroendocrine tumors. HIF-2α has been shown to be the driving force in ccRCC (43). HIF activation can also be controlled through PI3K/PTEN/AKT and RAS/RAF/MAPK signaling pathways. The former regulates the mTOR signaling that increases the translation of HIF protein, as described earlier. The latter has been reported to promote the HIF-coactivator p300 complex and subsequent transcription of target genes (42, 44, 45).

The metabolic components have also been shown to affect HIF stabilization through oxygen-independent regulation of PHD activity. The citric acid cycle (CAC) intermediate 2-OG is an absolute requirement for the enzymatic activity of PHD enzymes, whereas excess amount of other CAC metabolites fumarate, succinate, and oxaloacetate competitively inhibit PHDs (46). Inactivating mutations in enzymes processing fumarate and succinate leading to their accumulation, have been shown to induce HIF, and are associated with rare hereditary cancers, such as papillary renal cell carcinoma and paraganglioma (47–49). Mutations driving abnormal activation of isocitrate dehydrogenase (IDH) 3a were shown to decrease 2-OG amount causing HIF stabilization in cancer cells (50). Other types of IDH mutations lead to the production of 2-hydroxyglutarate (2-HG), which can competitively inhibit PHDs or even increase the activity depending on the 2-HG stereoisomerism (51, 52). However, the mechanisms for the PHD activation by 2-HG is debated (53). In triple negative breast cancer cells, the xCT glutamate-cystine antiporter has been shown to be inhibited by increased glutamate secretion. This in turn decreases the cellular cysteine level. Free cysteine prevents the oxidation of the specific cysteine residues in PHD2, but when the level decreases it leads to self-inactivation of PHD2 and subsequent HIF stabilization (54). Cancer cell-secreted lactate from glycolysis was shown to stabilize HIF in tumor-associated macrophages (TAMs) leading to induction in VEGF and arginase 1 (ARG1), markers associated with an M2 phenotype (55).

Multiple reports have showed that HIFs in cancer cells play a role in tumor progression. Inactivation of either HIF-1α or HIF-2α in *in vivo* breast cancer cell models decreases tumor growth and metastasis to the axillary lymph nodes (56), lung (57–61), and bone (62, 63). Hypoxia promotes cancer cell survival in multiple different ways and these have been reviewed in many papers (64, 65) These ways include the switch to anaerobic metabolism and neovascularization, which will be discussed further below. Hypoxia also promotes growth factor signaling, epithelial–mesenchymal transition, decrease in the apoptotic potential and evasion from the immune system. Cancer cells secrete growth factors to promote their own growth and survival as they generally also have membrane receptors for these factors, resulting in autocrine signaling. These growth factors also stimulate the surrounding stromal cells to change into a tumor promoting phenotype (65). HIF-1 has been shown to directly inhibit apoptosis by decreasing for instance the expression of the proapoptotic Bcl-2 family protein Bid (66) and to induce the expression of the apoptosis inhibitor survivin (67). In this way, cancer cells are protected from the harsh hypoxic environment leading also to a decrease in drug responsiveness (66). Hypoxia also modifies the cell surface proteins, which can shield the cells from immune system in many ways. For example, HIF-1 has been shown to directly upregulate programmed death-ligand 1 (PD-L1) (68) that suppresses T cell activation and CD47, which prevents phagocytosis by macrophages (69). HIF-1 induces a set of microRNAs that are small, non-coding RNA molecules known as hypoxamiRs (70). One of these is miR-210, which has been shown also to regulate cancer cell sensitivity toward cytotoxic T lymphocyte (CTL)-mediated lysis. miR-210 promotes cancer cell immune evasion without affecting cell surface proteins or CTL reactivity (71). In addition, HIF-induced miR-210 was shown also to promote angiogenesis. In cancer cells, miR-210 inhibits the expression and secretion of fibroblast growth factor receptor-like 1, which in turn was found to be a negative regulator of the angiogenesis (72).

In addition, hypoxia promotes cancer cell invasion and metastasis. For example, hypoxia enhances the motility and invasiveness of the cancer cells by promoting epithelial to mesenchymal transition. This happens by upregulating the expression of the transcription factors Snail1, Snail2, and Twist that downregulate expression of E-cadherin that is an important component of the adherens junctions. HIF also mediates this indirectly *via* Notch signaling (59, 73). Generally, CAFs are responsible for the remodeling of the ECM that assists in cancer cell invasion. However, numerous publications have proved that hypoxia induces cancer cells to secrete ECM remodeling enzymes that have essential roles in invasion, metastasis and premetastatic niche formation (60, 74–80). HIF-1 also mediates expression levels of angiopoietin-like 4 and L1 cell adhesion molecule. The former hampers vascular permeability and the latter cancer cell adhesion to the vasculature. Both are needed in intravasation and extravasation during the metastatic process (57).

Hypoxia also promotes the cancer stem cell phenotype. These cells are capable of unlimited cell division, differentiation to other cell types and, most importantly, can initiate cancer (59, 81). The hostile hypoxic microenvironment in the primary tumor primes the cancer cells to survive in the metastatic organ. There is some evidence that hypoxia plays a role in setting a dormancy phenotype by upregulating the main dormancy genes (NR2F1, DEC2, and p27) in breast cancer cells, which persist post-hypoxia helping the cancer cells to become therapy resistance (82).

Experiments have also been performed targeting PHDs that regulate HIF stability. Cancer cell-specific PHD2 haplodeficiency in the MMTV-PyMT breast cancer model led to increased HIF-1α and HIF-2α stabilization, but that did not have an effect on tumor growth or directly on the cancer cells’ invasive behavior. However, reduced lung metastasis was seen (83). In the MDA-MB-231 breast cancer model, PHD2 inactivation in cancer cells leads also to attenuate tumor growth. This resulted from decreased transforming growth factor β (TGF-β) processing leading to decreased expression of the extracellular protein osteopontin (SPP1), which has been associated with breast cancer malignancy (84). However, opposing results have been reported with the MCF-7 breast cancer model where PHD2 inactivation in cancer cells promoted tumor growth (84, 85) by upregulation of interleukin-8 (IL-8), VEGF, and the growth factor amphiregulin leading to increased vasculature formation (85). PHD2 inactivation led to HIF-1α stabilization and increased tumor growth in human colon carcinoma (HCT116), colorectal carcinoma (HT29 and RKO), and pancreatic carcinoma (SU.86.86) models. Generally, this was due to increased number of blood vessels. Further experiments with HCT116 cells revealed that the phenotype was not only HIF dependent. PHD2 was also shown to negatively regulate the transcription factor NF-κB. Hence, PHD2 inactivation led to increased secretion of angiogenic factors IL-8 and angiogenin (ANG) *via* NF-κB. These recruited bone marrow-derived cells (BMDCs) and promoted angiogenesis (86). PHD2 inactivation in murine osteosarcoma LM8, Lewis lung carcinoma (LLC), and B16BL6 melanoma cell lines also decreased tumor growth *in vivo*. The effect was claimed to be mainly HIF-independent since inactivation of HIF-1α led to increase in the tumor growth, which could be abolished when HIF-1α was inactivated together with PHD2. After more validation in the LM8 cell line, the PHD2 inactivation was shown to increase the number of vessels in the tumor but with decreased number of circulating cancer cells leading to reduced lung metastasis. LM8 cells were found to have increased expression of matrix metalloproteinases MMP2 and MT1MMP, which increased TGF-β signaling leading to decreased cancer cell proliferation (87, 88). PHD2 overexpression in the pancreatic cancer cells MIA PaCa-2 and PANC-1 decreased tumor growth by suppressing tumor vasculature in an HIF-dependent way resulting from decreases seen in VEGF and angiopoietin-1 (ANGPT1). However, this did not affect metastasis (89).

HIF prolyl hydroxylase 1 has been shown to play a role in cancer progression, but mainly in HIF-independent ways. PHD1 inactivation in ZR75-1, T47D, and MCF7 breast carcinoma cell lines decreased the tumor growth *in vivo* by HIF independently decreasing the cyclin D levels that are involved in cell cycling processes (90). Later PHD1 was shown to hydroxylate Forkhead box O3 (FOXO3), which hampers its interaction with the USP9x deubiquitinase leading to degradation. Decrease in FOXO3a decreases the cyclin D levels (91). Inhibition of PHD1 in HCT116 human colon carcinoma cells *in vitro* sensitized the cells for chemotherapy by decreasing p53 activation during the treatment and inhibited DNA repair increasing cell death (92).

## HIF and PHDs in Tumor Stroma: Good Cops?

It has become more evident that cancer progression is not only regulated by the cancer cells but also by the surrounding cancer stroma (93). In addition to the cancer cells, the TME includes different cells such as CAFs, endothelial cells (ECs), immune cells, growth factors, cytokines, and ECM (94). As described earlier, HIF activation in cancer cells has generally been regarded as a tumor-promoting factor, but opposing results have been shown with stromal cells. Global PHD2 haplodeficiency leading to increased stromal HIF-1α and HIF-2α stabilization did not have an effect on primary tumor growth in LLC and pancreatic carcinoma tumor models as well as in the MMTV-PyMT breast cancer metastasis model. Surprisingly, PHD2 haplodeficiency led to decreased lung metastasis, and this clearly demonstrated the importance of stromal hypoxic signaling on tumor progression (83, 95). Recent publications show that stromal factors together regulate the growth and metastatic capabilities of the cancer cells in a complex manner, but nevertheless revealing possible insights into new therapeutic targets.

## Hypoxic Response of CAFs

Cancer cells are known to harness normal quiescent fibroblast to promote cancer progression and one way they achieve this is by secreting TGF-β. This activates the fibroblasts that become CAFs with increased capacity to contract and remodel the ECM (96). CAFs are the most abundant stromal cell type and are generally thought to promote cancer progression. Many coculture experiments with CAFs and cancer cells show increased tumor growth when compared with normal fibroblast together with cancer cells (97). CAFs can promote cancer cell invasion by secreting growth factors and cytokines or remodeling the ECM—either making tracks or aligning fibrils for cancer cells to travel along (98). When fibroblasts become CAFs, their gene expression changes, and one of the most commonly used marker for CAFs is α-smooth muscle actin (αSMA). In addition to αSMA, CAFs have been shown to express other markers such as fibroblast-specific protein 1 (FSP1), platelet-derived growth factor receptor (PDGFR), fibroblast activation protein, and periostin, for example. Recently, it has become clear that CAF populations are heterogeneous with diverse markers, which may give them specific functions during tumorigenesis (99).

HIF-1 has been shown to play a role in normal fibroblast and CAF behavior. One experiment showed HIF-1α as a tumor suppressor when its inactivation in FSP1-expressing cells in MMTV-PyMT transgenic mice led to enhanced tumor growth by reducing the tumor vascular density with less leaky vessels together with decreased infiltration of TAMs. The molecular mechanisms behind these results were not characterized. No difference was seen, however, in tumor growth in the same model with HIF-2α-deficient fibroblasts (100). Another experiment with *in vitro* data indicated that hypoxic CAFs are involved in formation of new blood vessels in a manner requiring both HIF-1α and G-protein estrogen receptor to upregulate VEGF in hypoxia (101). HIF-1α has been shown to work as a tumor promoting factor as well. Experiments using the active form of HIF-1α in human skin fibroblast showed increased tumor growth when co-injected together with MDA-MB-231 cells, whereas HIF-2α did not have an effect. It was suggested that HIF-1α activation led to autophagy and aerobic glycolysis, which would produce nutrients to surrounding cancer cells promoting their growth (102, 103). PHD2 modulation in CAFs also affects tumor progression, but mainly *via* HIF. Hypoxia, PHD2 depletion, or PHD2 haplodeficiency was shown to inactivate CAFs by reducing their contraction and ECM remodeling capabilities (83, 104). Yes-associated protein 1 oncoprotein has been demonstrated to be important for CAF activation. It mediates the contraction *via* myosin light chain (MLC), which in turn regulates the contractile actomyosin function (105). Hypoxia and dimethyloxalylglycine (DMOG) decreased the levels of the phosphorylated MLC in CAFs (104). CAF inactivation was further demonstrated *in vivo* when CAFs with either PHD2 haplodeficiency or shRNA inactivation were orthotopically transplanted together with breast cancer cells leading to decreased lung metastases but without any changes in primary tumor growth (83, 104). Also, systemic PHD inhibition with DMOG decreased lung and liver metastases in a 4T1 breast cancer model. DMOG treatment also reduced the stiffness and αSMA levels within the tumors without affecting the number of αSMA positive cells (104). However, PHD2 haplodeficiency in platelet-derived growth factor receptor α (BDGFRα)-positive CAFs in MMTV-PyMT transgenic mice did not have an effect on the lung metastases (83). As a side note, targeting only one CAF marker might not ensure wide inactivation in a heterogeneous CAF population (99).

## Hypoxic Response of the Tumor Vasculature

The tumor vasculature is essential in supplying nutrients and oxygen to the fast dividing cancer cells but becomes quickly inadequate to meet the need of a fast expanding tumor. Oxygen deprivation in the tumor causes HIF stabilization and upregulation of proangiogenic factors both from cancer cells and tumor stromal cells to promote the vessel formation needed for tumor progression and metastasis (106). ECM proteins have also been shown to be involved in angiogenesis. Hypoxia-inducible LOX promotes the expression of VEGF in cancer cells *via* PDGFRβ-mediated Akt activation (107). Tumor vasculature, however, has been found to be abnormal with morphological changes, leakiness, and aberrant pericytes, which result in poorly perfused vessels that are not able to rescue the hypoxic condition. This results from excessively secreted pro- and antiangiogenic factors leading to disorganized vessel formation. This can hinder, for example, the delivery of anticancer drugs and also benefit cancer cells during their extravasation process (108).

Inactivation of HIF-1α in Tie2 positive ECs lead to decreased tumor growth in a subcutaneous LLC model, caused by reduced density of tumor vessels. ECs had decreased proliferation and migration under hypoxia as well as decreased expression levels of VEGF and VEGF receptor-1 (VEGFR-1). This demonstrated the importance of EC autocrine expression of these factors during vessel formation (109). Similar results were obtained with the MMTV-PyMT breast cancer model and the LLC model. HIF-1α inactivation in Tie2-positive ECs drastically decreased lung metastasis, but only had moderate effect on primary tumor growth. In addition, HIF-1α inactivation decreased the expression of inducible nitric oxide synthase (iNOS), further decreasing the amount of nitric oxide (NO) and leading to less permeable ECs for cancer cell migration. HIF-2α inactivation in ECs on the other hand led to decreased levels of arginase 1 (ARG1), which in turn increased the NO levels making the EC layer more accessible for cancer cells. Supporting this finding, *in vivo* tail vein injection of LLC cells showed more cancer cell colonization in the lung of HIF-2α EC null mice (110). However, HIF-2α inactivation from vascular endothelial (VE)-cadherin positive ECs decreased tumor growth in LLC and B16F1 models, as well as in carcinogen-induced skin epithelial tumors. HIF-2α inactivation was shown to increase the number of vessels and their branching but decreasing the number of mature functional tumor vessels. This resulted from disrupted delta-like ligand 4/Notch (DII4/Notch) signaling leading to decreased expression of angiopoietin-2 (111, 112). These results show different roles for HIF-1 and HIF-2 in angiogenesis. Mice with PHD2 haplodeficiency in Tie2-positive ECs did not have an effect on the primary tumor growth, but the amount of liver metastases in a pancreatic cancer model was reduced. This resulted from HIF-2-induced expression of sVEGFR-1 and VE cadherin that normalized ECs with less permeable vessels leading to a less hypoxic tumor without any changes in the actual number of vessels (83, 95).

Pericytes are mural cells that cover the ECs and provide vascular stability and are known to signal to each other during angiogenesis (108). Pericyte depletion was shown to disrupt primary tumor growth by decreasing the microvessel density and increasing hypoxia, but this, however, led to increased metastasis showing their importance in tumor vasculature (113). Later, the increased hypoxia was shown to induce Ang-2 expression within the tumor which resulted in vascular instability and lung metastasis. This could be restored by inhibiting Ang-2 signaling (114).

## Hypoxic Response of Immune Cells

The microenvironment of solid tumors also contains tumor infiltrating immune cells. These include lymphoid cells and myeloid cells, such as T- and B-cells and natural killer cells, TAMs, myeloid-derived suppressor cells (MDSCs), and neutrophils, which all have both pro- and antitumorigenic effects. Hypoxia has been shown to alter immune resistance and suppression, which helps tumor cells to survive immune surveillance (2). TAMs are the most common immune cells found within the tumor and are generally associated with tumor progression and also have been shown to promote cancer cell invasion and angiogenesis (115). Hypoxic tumor regions have been shown to recruit TAMs *via* VEGF and semaphoring 3A expression and once in a low oxygen condition their gene expression profile changes to a more cancer promoting phenotype (116, 117). Macrophages are known to have plasticity, and their phenotype can change based on external cues. Once they get activated they can have tumor suppressing M1 or tumor promoting M2 polarization. Stimulation driving the M1 phenotype also activates HIF-1 which upregulates iNOS levels and drives NO synthesis. During M2 polarization HIF-2 is induced leading to an increase in arginase-1, which in turn decreases the NO synthesis (118). *In vitro* assays suggest that depletion of HIF-1α in TAMs induces more M2 phenotype (119). By using PHD2-haplodeficient mice, which ensured less hypoxic tumors, Laoui et al. showed, however, that the oxygenation state in the tumor did not significantly affect the differentiation of TAMs into M1 or M2 like (120). They saw changes in hypoxia-regulated genes in M2 like TAMs, but not in M1 like TAMs. Overall the results suggest that hypoxia itself does not influence the activation of the TAMs into M1 nor M2 (120).

HIF-1α- and HIF-2α-expressing macrophages have been shown to promote tumor progression. Inactivation of HIF-1α from lysozyme 2 (LysM)-positive neutrophils and macrophages in MMTV-PyMT mice led to hindered tumor progression and smaller tumors. This was shown to result from an increased number of cytotoxic T-cells (121). Mice lacking HIF-2α from LysM-positive macrophages in inducible hepatocellular carcinoma and colitis-associated cancer had reduced number of TAMs in tumor regions which associated with a delayed tumor progression tendency (122). However, opposing results have been shown with PHD inactivation. One experiment showed that PHD2 inactivation in macrophages together with CD4^+^ T-cells decreased the tumor progression by overall downregulation of protumoral and antitumoral cytokines eventually leading to increased tumor cell death in an LLC model, and this was in part because of HIF-1α stabilization (123). Mice with a melanoma tumor model were treated with granulocyte–macrophage colony-stimulating factor together with systemic inhibition of PHD3 by AKB-6899 leading to decreased tumor size and lung metastasis. This resulted from HIF-2α stabilization in TAMs, which increased the expression of VEGF sequestering the soluble VEGFR-1 which decreased the vasculature within the tumor (124).

Hypoxia and HIF-1α stabilization regulates the MDSCs within the tumor enhancing their role as T-cell suppressors (125). Tumor-infiltrating MDSCs, as well as macrophages and tumor cells, were shown to have increased levels of the cell surface protein PD-L1, which was shown to be an HIF-1α target and upregulated by hypoxia. PD-L1 upregulation in MDSCs suppressed T-cell activation, whereas PD-L1 blockade under hypoxia enhanced MDSC-mediated T-cell proliferation and function and was associated with decreased interleukin-6 and interleukin-10 expression (68). Hypoxia has been shown to decrease the expression of the cell surface protein major histocompatibility complex class I *in vivo* and *in vitro* in renal cell carcinoma cells providing them a mechanism to evade immune surveillance by cytotoxic T-cells (126).

## Hypoxic Response of the ECM

In addition to the different cell types within the TME, the ECM composition and structure also contributes to tumor progression (94). The ECM does not only provide an architectural scaffold defining tissue boundaries but it also regulates cellular behavior, such as cell adhesion and migration by mechanical and biochemical cues. The ECM can bind and store growth factors or directly interact with receptors on the cell surfaces. The ECM is a dynamic meshwork of proteins that is constantly being remodeled and it is rich in proteoglycans and fibrillar collagens. ECM proteins have also been shown to directly induce cancer cell proliferation and metastasis. LOX is upregulated by hypoxia, and in a colorectal cancer model, LOX induced cancer progression *via* activation of SRC signaling (127). The ECM is mainly secreted by CAFs as it is one of the main functions of fibroblast to maintain the homeostasis of the ECM. CAFs, however, have abnormal ECM regulation activity, and they have been shown to secrete more ECM proteins than normal fibroblasts (98). In addition to CAFs, cancer cells are known to play a part in ECM remodeling (128). A high collagen content together with a stiff ECM is generally associated with malignant metastatic cancers (75, 129). The biosynthesis of the fibrillar collagens requires posttranslational modifications that take place both inside and outside the cell with the help of collagen modifying enzymes, many of which are known to be regulated by HIF (130). Collagen modifying enzymes have been shown to have important roles in cancer progression. Inactivation of collagen prolyl 4-hydroxylase P4HA1 or P4HA2 in MDA-MB-123 breast cancer cells decreased collagen deposition and attenuated tumor growth and resulted in as much as 99% decrease in lung metastasis (79). Inactivation of lysyl hydroxylase PLOD2 in the same cancer model decreased the metastasis to lungs resulting from inefficient formation of collagen fibers and decreased tumor stiffness (78). Inhibiting LOX and lysyl oxidase-like 2 (LOXL2) and LOXL4 decreased metastasis in several cancer cell types (60, 74, 76). LOX, LOXL2, and LOXL4 deficiencies decreased collagen cross-linking and stiffness which was also needed to recruit CD11b^+^ BMDCs into the premetastatic niches to promote tumor cell colonization (60, 77, 80). LOXL2 in turn upregulated the expression of matrix remodeling enzymes such as tissue inhibitor of metalloproteinase-1 and matrix metalloproteinase-9 (MMP9) (76). LOXL2 also activated CAFs *via* integrin β3 and promoted their collagen contraction, a phenomenon known to increase matrix stiffness (131).

Cancer-associated fibroblasts are able to mechanically remodel the ECM by active contraction resulting in matrix stiffening (105). CAFs with PHD2 haplodeficiency were less active than wild-type CAFs which was demonstrated by decreased expression levels of markers associated with CAFs (αSMA, FSP1, and PDGFRα) and decreased contraction capabilities (83). PHD2 haplodeficiency in CAFs also decreased matrix production, and this was accompanied by decreased expression of collagen modifying enzymes LOX, P4HA1, P4HA2, PLOD2 as well as matrix metalloproteinases MMP2 and MMP9. This is partly surprising, since hypoxic fibroblast and cancer cells generally upregulate the expression of the same genes (132). Similar results were seen with human head and neck carcinoma-associated fibroblasts and human vulvar carcinoma-associated fibroblasts. PHD2 inactivation or 1% oxygen decreased αSMA expression, and they had decreased contraction capabilities (104).

## Hypoxic Response of Cancer Metabolism

One of the most common characteristics for cancer cells is rapid proliferation, which is accompanied by high demands of energy. In normoxia, cells generally produce energy as ATP by mitochondrial oxidative phosphorylation, which is an O_2_-dependent pathway. As tumors grow, cells face the limitation of decreased oxygen concentration and switch to inefficient glycolysis to ensure ATP production (46). HIF-1 has been shown to be an important regulator in the metabolic switch as it induces the expression of genes that adjust the cellular metabolism away from oxidative phosphorylation toward increased glycolysis. This mechanism is not just to ensure the ATP production under hypoxia but also to prevent excessive formation of reactive oxygen species (ROS). Most importantly HIF-1 induces pyruvate dehydrogenase kinase 1 that inhibits the activity of the pyruvate dehydrogenase complex (PDC). PDC regulates the first step needed in oxidative phosphorylation where pyruvate is converted to acetyl coenzyme A (133). HIF-1α also upregulates lactate dehydrogenase A and monocarboxylate transporter 4 which promotes conversion of pyruvate to lactate (end product of glycolysis) and transports it out of the cell, respectively (134, 135). HIF activation also upregulates the expression of glucose transporters GLUT1 and GLUT3, which are responsible for the glucose import into the cells (136, 137). Lactate from glycolysis is considered as a major reason for the tumor acidification. Hypoxia upregulates carbonic anhydrase IX, which regulates the tumor pH and has an important role in the survival of tumor cells in hypoxic regions of tumors and metastasis (138). In addition, HIF indirectly targets the metabolic genes that contribute to the glycolytic shift by upregulating miR-210. miR-210 in turn inhibits multiple targets such as iron-sulfur cluster assembly proteins (ISCU), cytochrome *c* oxidase assembly protein (COX10), NADH dehydrogenase (ubiquinone) 1 alpha subcomplex 4 (NDUFA4), and succinate dehydrogenase complex subunit D (SDHD), all of which play a role in mitochondrial function (139–141). PHD3 was in turn shown to induce the activity of PDC HIF independently by binding to the complex. PHD3 deficient cells were seen to have clear decreased PDC activity, which promoted survival in prolonged hypoxia together with a lower amount of ROS (142). HIF also suppresses mitochondrial function by downregulating multiple components in the electron transport chain and also by direct mitochondrial autophagy mediated by increased BCL2 interacting protein 3 (BNIP3) levels. In this way, hypoxia also decreases the levels of ROS, which are harmful in increased quantities (143). There are reports stating that mitochondrial ROS itself also regulates HIF, but there is also many opposing results [reviewed in Ref. (144)]. Increased ROS levels have been shown to promote HIF stabilization during hypoxia, by further inhibiting PHD enzymes. Contradicting results claim it is not ROS regulating HIF stabilization, but rather the mitochondria, which monitor the intracellular oxygen availability. Under normoxic conditions ROS are able to stabilize HIF, but the exact mechanism is not clear. It has been suggested to be *via* inhibition of PHDs or pVHL, but another signaling pathway has been proposed. HIF-1α gene expression was shown to be upregulated in oral squamous cell carcinoma and MCF-7 breast cancer cells by ROS *via* induced ERK and P13K/AKT signaling (145, 146). Overall, HIF-regulated glucose metabolism supplies sufficient glucose import for less efficient glycolysis and removes the excess end products out of the cell. It defends the cells against increased ROS production, which is generated in the oxidative phosphorylation pathway under too low oxygen concentration and gives the cancer cells protection to proliferate and grow under hypoxic conditions (147).

Within the solid tumor there are regions that are not under hypoxia, but still the cancer cells have a tendency to switch to inefficient glycolysis even in presence of oxygen. However, the metabolic state in the different cells within the tumor is heterogeneous and there has been evidence of supplying metabolites between cells (148). When fibroblasts become CAFs, they have been shown to decrease the expression of isocitrate dehydrogenase 3α. This leads to a decreased amount of effective 2-oxoglutarate, which is needed as a co-substrate in the hydroxylation reaction, and increasing levels of succinate and fumarate. This results to PHD inhibition, which stabilizes HIF-1α and promotes glycolysis in the CAFs. Metabolites from the normoxic glycolysis in CAFs was hypothesized to feed the surrounding cancer cells (149).

## Conclusion and Future Perspectives

**Figure 2:**
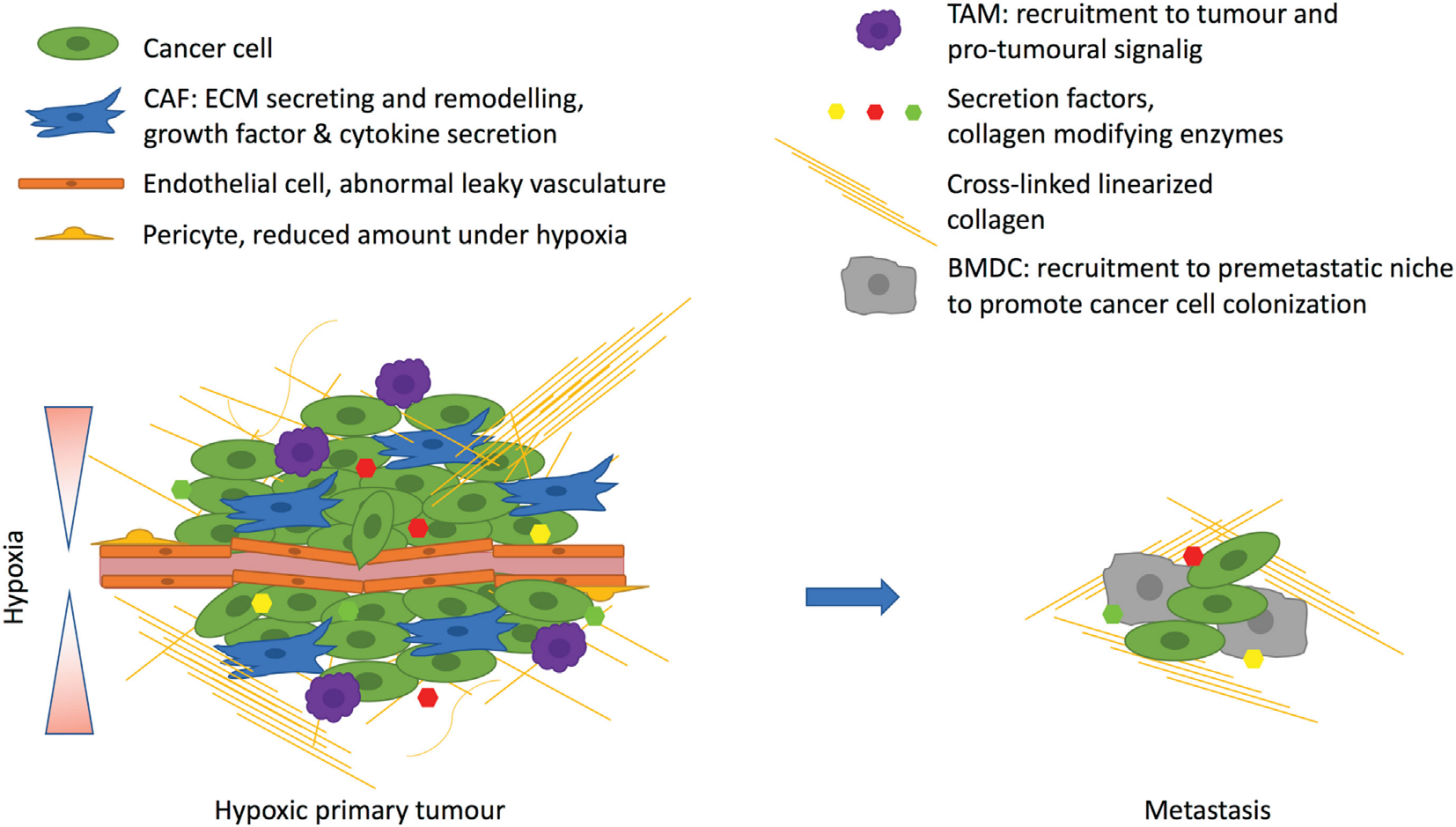
Hypoxia affects the tumor microenvironment. Hypoxia arises due to rapidly proliferating cancer cells with inadequate vasculature. The excessive angiogenic signaling promotes formation of abnormal leaky vasculature with a decreased amount of pericytes. Hypoxia promotes tumor-associated macrophage (TAM) infiltration, which support tumor growth. Hypoxic cancer cells corrupt fibroblasts by secreting, for example, transforming growth factor β, which turns fibroblast into cancer-associated fibroblasts (CAFs) to drive the cancer progression. CAFs promote desomplasia and together with cancer cells they secrete extracellular matrix (ECM) components such as collagens. One important secreted factor is the hypoxia-inducible enzyme lysyl oxidase, which cross-links fibrillar collagen at the primary tumor and prepares the premetastatic niche for the recruitment of bone marrow derived cells (BMDCs) that are needed for cancer cell colonization at the metastatic site.

Hypoxia is a condition that plays a role in normal physiology, such as during embryonic development, but it is more commonly associated with pathological states. Oxygen deprivation is a common feature of all solid tumors and affects all components of the TME (Figure 2). Hypoxia has clear effects on both cancer cells and surrounding stromal cells, but it does not promote tumor progression in every case. Different cell types seem to be differentially regulated by the hypoxic environment. In cancer cells, HIF stabilization generally promotes tumor progression. Inactivation of HIF-1 or HIF-2 in cancer cells in mouse cancer models decreases the tumor growth and the formation of metastases in many ways, practically affecting all the hallmarks of cancer. When cancer cells are targeted with PHD2 inactivation, which enables HIF stabilization, the results are more variable. PHD enzymes do also have HIF-independent targets and functions that can complicate their role in cancer promotion. However, PHD2 haplodeficiency in ECs leads to less tumor hypoxia resulting from HIF-2 activation in the ECs themselves stimulating EC intrinsic proangiogenic signaling leading to EC normalization. Global haplodeficient inactivation of PHD2 or treatment with a PHD inhibitor does not have an effect on primary tumor growth, but it rather decreases metastasis. Targeting PHD2 in CAFs leads to their deactivation and reduces their capabilities to promote cancer cell invasion and metastasis both *in vitro* and *in vivo*. These findings suggest that global targeting of PHD and HIF activities is sufficient to reprogramme the TME to suppress tumor progression. However, we are only beginning to understand how hypoxia, HIFs and PHDs alter the TME, and further research is required to gain insight. Thus, while hypoxia research has been performed for decades, recent data suggests that there is much more to learn, and that this may have important clinical implications regarding the use of agents that target hypoxia pathways such as HIF or PHD inhibitors.

## Author Contributions

The review was written by AL and edited by JTE.

## Conflict of Interest Statement

The authors declare that the research was conducted in the absence of any commercial or financial relationships that could be construed as a potential conflict of interest.
